# Influenza and respiratory syncytial virus dynamics in Lao PDR during the COVID-19 pandemic: a hospital-based surveillance study

**DOI:** 10.1136/bmjopen-2024-098006

**Published:** 2025-09-04

**Authors:** Koukeo Phommasone, Danoy Chommanam, Nathaniel C Christy, Touxiong Yiaye, Soulichanya Phoutthavong, Patsalin Keomoukda, Sompong Thammavong, Thipsavanh Bounphiengsy, Thongsavanh Lathsachack, Latsaniphone Boutthasavong, Vayouly Vidhamaly, Bountoy Sibounheuang, Ooyanong Phonemixay, Siribun Panapruksachat, Viladeth Praphasiri, Sommay Keomany, Bounthavy Chaleunphon, Phouvieng Douangdala, Matthew T Robinson, Elizabeth M Batty, Manivanh Vongsouvath, Michael R Wiley, Jessica D Wiley, Ryan C Chapman, Andrew G Letizia, Mayfong Mayxay, Audrey Dubot-Pérès, Elizabeth A Ashley

**Affiliations:** 1 Microbiology Laboratory, Lao-Oxford-Mahosot Hospital-Wellcome Trust Research Unit, Mahosot Hospital, Vientiane, Lao People’s Democratic Republic; 2 U.S. Naval Medical Research Unit INDOPACIFIC, Singapore; 3 Xiengkhouang Provincial Hospital, Phonsavan District, Xiengkhouang Province, Lao People’s Democratic Republic; 4 Salavan Provincial Hospital, Salavan Province, Lao People’s Democratic Republic; 5 Attapeu Provincial Hospital, Attapeu Province, Lao People’s Democratic Republic; 6 Luang Namtha Provincial Hospital, Luang Namtha Province, Lao People’s Democratic Republic; 7 Nuffield Department of Clinical Medicine, Centre for Tropical Medicine and Global Health, University of Oxford, Oxford, UK; 8 Mahidol-Oxford Tropical Medicine Research Unit, Faculty of Tropical Medicine, Mahidol University, Bangkok, Thailand; 9 PraesensBio, LLC, Omaha, Nebraska, USA

**Keywords:** COVID-19, Respiratory infections, SARS-CoV-2 Infection

## Abstract

**Abstract:**

**Objectives:**

Globally, the circulation of influenza and other seasonal respiratory viruses changed dramatically during the COVID-19 pandemic. This study aims to determine the trends of acute respiratory infections (ARIs) caused by SARS-CoV-2, influenza A, influenza B and respiratory syncytial viruses (RSVs) in patients presenting to hospitals in the Lao People’s Democratic Republic (PDR) (Laos).

**Design:**

Prospective surveillance study.

**Setting:**

Four provincial hospitals across Laos between March 2021 and July 2023.

**Participants:**

Participants of all ages who met our case definition for an ARI (axillary temperature ≥37.5°C or history of fever AND cough or other respiratory symptoms/signs OR loss of smell and/or taste) presenting to the hospital less than 10 days after symptom onset were eligible to be enrolled in the study. Combined nasopharyngeal and throat swabs were tested for SARS-CoV-2, influenza A, influenza B and human RSV (hRSV) using probe-based real-time RT (Reverse transcription)-PCR assays.

**Primary outcome measure:**

The proportion of patients in whom SARS-CoV-2, influenza A, influenza B and hRSV was detected.

**Results:**

There were 4203 patients recruited, of whom 898 (21%) were children aged under 5 years. SARS-CoV-2 was detected in 16.9% of patients, followed by influenza A, influenza B and hRSV (8.4%, 7.2% and 4.7%, respectively). 98 patients (2.3%) were diagnosed with probable co-infection, with at least two viruses detected. After May 2022, the number of cases of influenza A, influenza B and hRSV increased rapidly. Six per cent of patients (263) had a quick Sequential Organ Failure Assessment score of ≥2, and 34 (0.8%) patients died, of whom 11 tested positive for a respiratory virus.

**Conclusions:**

During the COVID-19 pandemic in Laos, few respiratory viruses were detected by passive surveillance until the relaxation of non-pharmaceutical interventions implemented for infection control. After restrictions were lifted, influenza A, influenza B and hRSV emerged rapidly, showing the importance of continuous surveillance.

**STRENGTHS AND LIMITATIONS OF THIS STUDY**

- The strength of this study was its ability to track fluctuations in SARS-CoV-2 and other respiratory viruses during the early stages of the pandemic in Laos.
- Limitations of this study include our convenience sampling approach at the provincial hospital level, which prevented us from making accurate age-related incidence estimates.
- Our testing strategy included only four viruses and no bacteria.

## Background

 Circulation of influenza and other seasonal respiratory viruses changed dramatically during the COVID-19 pandemic, with an overall drop in incidence in the northern and southern hemispheres.^1^ The changing dynamics of respiratory infections have primarily been attributed to the control measures put in place to reduce transmission of SARS-CoV-2, such as masks, physical distancing, school closures, etc, with the possibility of viral interference also considered.^1^,^4^ The influenza B/Yamagata lineage has not been detected worldwide since the first quarter of 2020, leading to tentative conclusions that the COVID-19 pandemic likely led to its extinction.^5^ Numerous countries reported changes in the epidemiology of human Respiratory Syncytial Virus (hRSV), in some cases with disappearances during the early part of the pandemic. This was followed by atypical resurgences of transmission at different times from usual seasonal peaks and/or larger peaks of infections.^6^,^10^

The Lao People’s Democratic Republic (PDR) (Laos) is a country in the WHO Western Pacific Region that usually experiences peaks of influenza transmission from July to December.^11 12^ There are sparse data available on hRSV trends, with most reports coming from detection in patients in the capital, Vientiane, and only one study from Luang Prabang, located farther north, in 2019, which reported a single peak of hRSV during the rainy season (between May and December).^13 14^

Although respiratory infection data are limited in Laos, a previous study of patients with symptoms of acute respiratory infection (ARI) and age-matched and sex-matched community controls between June 2019 and June 2020 at one site in northeastern Laos attributed the aetiology of symptomatic infection to influenza B virus, influenza A virus, human metapneumovirus and hRSV in 17.8%, 17.2%, 7.5% and 6.5% of participants, respectively.^15^ Neither patients nor controls tested positive for SARS-CoV-2, using the same methods as in this study. We continued case-based surveillance at three sites across Laos under an approved protocol before this study began in March 2021 and screened a further 499 patients with respiratory symptoms between August 2020 and February 2021: 66 cases tested positive for hRSV, 6 for influenza A virus and none for influenza B virus and SARS-CoV-2 (LOMWRU, unpublished data).

PCR testing was established in Laos in February 2020. The first cases of laboratory-confirmed COVID-19 in Laos were reported on 24 March 2020.^16^ Following a nationwide lockdown from early April 2020, a national seroprevalence study conducted from August to September 2020 confirmed negligible SARS-CoV-2 circulation in the community at that time.^17^ Strict border controls and admission of cases to hospital or quarantine centres were observed until May 2022. Laos first received Sinopharm COVID-19 vaccines from China on 31 December 2020 and AstraZeneca from the COVAX facility on 20 March 2021.^18^ Mass vaccination was first provided to healthcare workers and frontliners in March 2021. From its launch until October 2022, primary series vaccination coverage was around 78% of the total population.^19^

Schools in Laos gradually began reopening at the end of January 2022, starting with Grade 5 of primary school and Grades 4 and 7 of secondary school. The WHO declared the end of COVID-19 as a Public Health Emergency of International Concern on 5 May 2022. Two days later, all international borders in Laos were reopened with no quarantine measures, and this was followed by the gradual relaxation of mask-wearing in all workplaces and public places.

We conducted prospective, case-based surveillance for respiratory virus infections in four provincial hospitals across the country between March 2021 and July 2023. The results of this study period are reported herein and demonstrate a unique perspective on the dynamics of respiratory viruses during the COVID-19 pandemic.

## Methods

### Study sites

**Figure 1:**
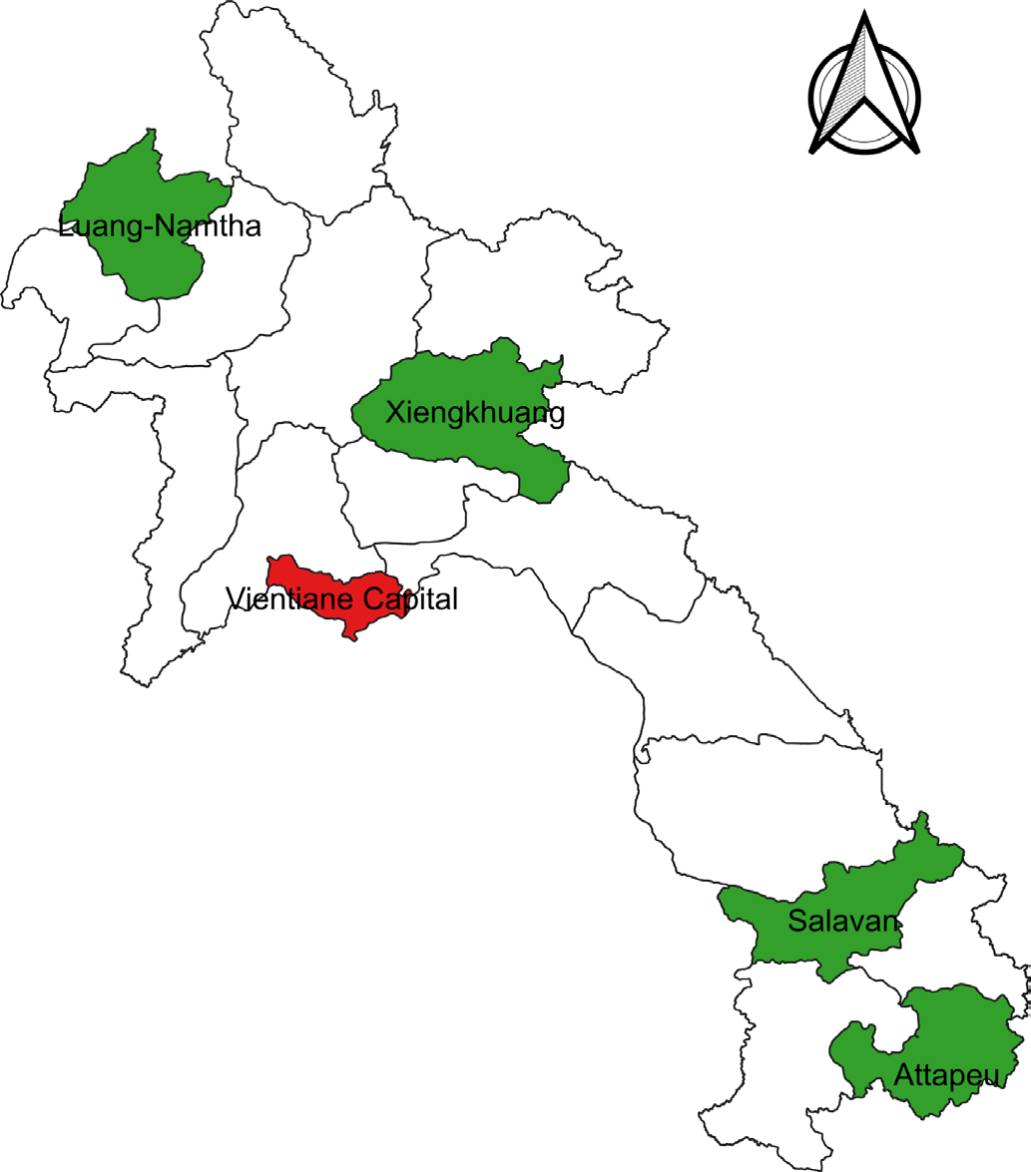
Map of the Lao People’s Democratic Republic. Study sites are indicated in green, and Vientiane Capital, where the central laboratory is located, is marked in red.

This surveillance study was conducted at four provincial hospitals, which serve as secondary care facilities: Luang Namtha (LNT) Provincial Hospital, a 60-bed hospital close to the Laos-China border; Xiengkhouang (XK) Provincial Hospital (100 beds) in northeastern Laos; Salavan (SV) Provincial Hospital (70 beds) and Attapeu (ATP) Provincial Hospital (100 beds), both located in southern provinces, close to the Laos-Cambodia and Laos-Vietnam borders (figure 1). This study was designed as a prospective case-based surveillance of outpatients and inpatients, and a formal sample size calculation was not performed.

Male and female participants of all ages who met our case definition for an ARI (axillary temperature ≥37.5°C or history of fever AND cough or other respiratory symptoms/signs OR loss of smell and/or taste) presenting to the hospital less than 10 days after symptom onset were eligible to be enrolled in the study, provided written informed consent was obtained from the participant or parent/guardian. Symptoms, medical history, vital signs and physical examination findings were documented using the tablet-based case-report form designed by the International Severe Acute Respiratory and Emerging Infection Consortium.^20^ Nasopharyngeal and throat swabs were taken using flocked swabs (FLOQSwab 516CS01, COPAN Diagnostics, Brescia, Italy) by a trained healthcare professional wearing appropriate personal protective equipment. Swabs were placed together in tubes containing virus transport medium, Sigma Virocult (1 mL until December 2022 in ATP, until January 2023 in LNT and until February 2023 in XK, and then 3 mL (Medical Wire and Equipment, Corsham, UK)). The samples were transferred to the laboratories of provincial hospitals for aliquoting and kept at −20°C before being shipped on dry ice on a weekly basis to Vientiane for real-time RT-PCR (RT-qPCR, Real-time quantitative polymerase chain reaction) testing. Between 10 and 25 eligible patients were recruited per week per site. Recruitment rate per month fluctuated depending on lockdown measures. The SV site was closed in June 2021, and the ATP site opened in February 2022. Recruited participants were followed up at day 28 (−2 days and +7 days) and their vital status was recorded.

The quick Sequential Organ Failure Assessment (qSOFA) was also used to identify patients with suspected infection who may be at risk of clinical deterioration. This universal screening tool is composed of three criteria: altered mental status, respiratory rate ≥22 breaths per minute and systolic blood pressure ≤100 mm Hg. A score of two or more suggests a higher risk of mortality.

### Real-time RT-PCR assays for virus detection and genome sequencing

Specimens were processed at the central laboratory in Mahosot Hospital, Vientiane, Laos. RNA extraction was performed using the EZ1 Virus Mini Kit (Qiagen), and then submitted to probe-based real-time RT-PCR (RT-qPCR) for the detection of SARS-CoV-2,^21^ influenza A virus,^22^ influenza B virus^23^ and hRSV,^24^ using conditions adapted from the corresponding publications. Whole genome sequencing of SARS-CoV-2 and influenza A virus was also performed at the same laboratory on samples found positive by the corresponding RT-qPCR with a Ct (cycle time)≤28. Details of the RT-qPCR and whole genome sequencing methods for SARS-CoV-2 and influenza A virus can be found in the online supplemental material.

### Data analysis

Statistical analysis was performed using STATA (Statacorp 2023) V.18.0. Data were presented as percentages, mean with SD, median with range or IQR depending on their distribution.

### Patient and public involvement statement

Patients were not involved in the design, reporting or dissemination plan of the research.

## Results

Between March 2021 and July 2023, 4203 patients were enrolled in this study (1437 in LNT, 1825 in XK, 125 in SV and 816 in ATP), with a mean (SD) recruitment rate of 145 (74) patients per month. Almost half of the participants reported receiving at least one dose of a COVID-19 vaccine, with half of these having received Sinopharm, followed by Johnson and Johnson, AstraZeneca, Sinovac, Pfizer and Sputnik. Most (3281; 78.1%) patients had mild or moderate disease (qSOFA<2) and were managed as outpatients (online supplemental table 1). 85% of the confirmed COVID-19 patients presented with a sore throat before the arrival of the Omicron variant in May 2022, while 79% of them presented with a sore throat during the Omicron variant surge (data not shown). Participant characteristics are shown in table 1. The majority (63.4%) were aged over 15 years, and 51% were male. The most commonly reported comorbidity among participants was hypertension, present in 5.8% of participants. Fewer than 1% of participants reported diabetes mellitus. Around 22.6% had already taken antibiotics before presenting to the hospital.

**Table 1:** Sociodemographic characteristics and comorbidities

Variables, n (%)	Total	LNT	XK	SV	ATP
	n=4203	n=1437	n=1825	n=125	n=816
Healthcare worker	56 (1.3)	6 (0.4)	16 (0.9)	0	34 (4.1)
Gender (male)	2130 (50.7)	721 (50.2)	905 (49.6)	73 (58.4)	431 (52.8)
Age group					
<1 year	258 (6.1)	129 (8.9)	77 (4.2)	3 (2.4)	49 (6.0)
1–5 years	640 (15.2)	316 (24.1)	181 (10.3)	24 (19.6)	119 (14.6)
5–15 years	640 (15.2)	210 (16.1)	187 (10.7)	62 (50.8)	181 (22.2)
>15 years	2665 (63.4)	782 (59.8)	1380 (78.9)	36 (29.5)	467 (57.2)
Previously known COVID-19 infection*	170 (5.4)	15 (1.3)	112 (9.4)	0	43 (5.3)
Vaccination COVID-19^†^	1827 (43.7)	435 (30.3)	867 (47.5)	7 (5.6)	518 (63.5)
Chronic heart diseases	40 (0.9)	16 (1.1)	20 (1.1)	0	4 (0.4)
Hypertension	248 (5.7)	46 (3.2)	157 (8.6)	3 (2.4)	42 (4.4)
Chronic pulmonary diseases	84 (1.9)	23 (1.6)	52 (2.8)	1 (0.8)	8 (0.8)
Asthma	98 (2.3)	9 (0.6)	63 (3.4)	1 (0.8)	25 (2.6)
Renal diseases	24 (0.5)	9 (0.6)	11 (0.6)	0	4 (0.4)
Diabetes	17 (0.4)	8 (0.5)	4 (0.2)	5 (4)	0
History of pulmonary TB	27 (0.6)	14 (1)	7 (0.4)	1 (0.8)	5 (0.5)
BMI					
Underweight	1194 (33.2)	460 (41.5)	365 (21.9)	82 (71.5)	286 (40.7)
Normal	1852 (51.5)	514 (46.3)	971 (58.3)	29 (25.0)	338 (48.1)
Overweight	475 (13.2)	117 (10.5)	286 (17.2)	4 (3.4)	68 (9.7)
Obesity I	55 (1.5)	9 (0.8)	37 (2.2)	0	9 (1.3)
Obesity II	10 (0.3)	2 (0.2)	6 (0.3)	0	2 (0.3)
Obesity III	8 (0.2)	7 (0.6)	1 (0.06	0	0
Current smoker	305 (7)	33 (2.3)	159 (8.7)	7 (5.6)	89 (10.9)
Antibiotics prior to hospital presentation	949 (22.6)	15 (0.01)	559 (30.6)	44 (35.2)	331 (40.6)

BMI for patients aged over 2 years was categorised as: underweight (BMI<18.5), normal (BMI=18.5–24.9), overweight (BMI=25–29.9), obesity I (BMI=30–34.9), obesity II (BMI=35–39.9) and obesity III (BMI≥40).

* Previous SARS-CoV-2 infection reported by patient.

† At least one dose of COVID-19 vaccine.

ATP, Attapeu; BMI, Body Mass Index; LNT, Luang Namtha; SV, Salavan; TB, tuberculosis; XK, Xiengkhouang.

### Clinical presentation and outcomes

Among 3939 patients, 108 (2.7%) presented with oxygen saturations of 90% or lower on room air. Using the qSOFA score, 263 (6%) participants met the criteria for suspected sepsis, and 45 (1%) patients (including 31 children) were admitted to the intensive care unit. 34 patients (0.8%), including 18 males, died; the median age was 58 years, with a range from 3 months to 86 years. Of those, 12 (0.3%) patients died in hospital, and 22 (0.5%) patients died at home within 28 days of hospital discharge. Among the patients who died, 10 (29%) had at least one respiratory virus detected in their samples. Six tested positive for SARS-CoV-2, one for SARS-CoV-2 and influenza A virus, one for SARS-CoV-2 and hRSV and two for influenza B virus. A description of the participants who died is in the supplementary material (online supplemental tables 2 and 3).

### Virus detection

**Figure 2:**
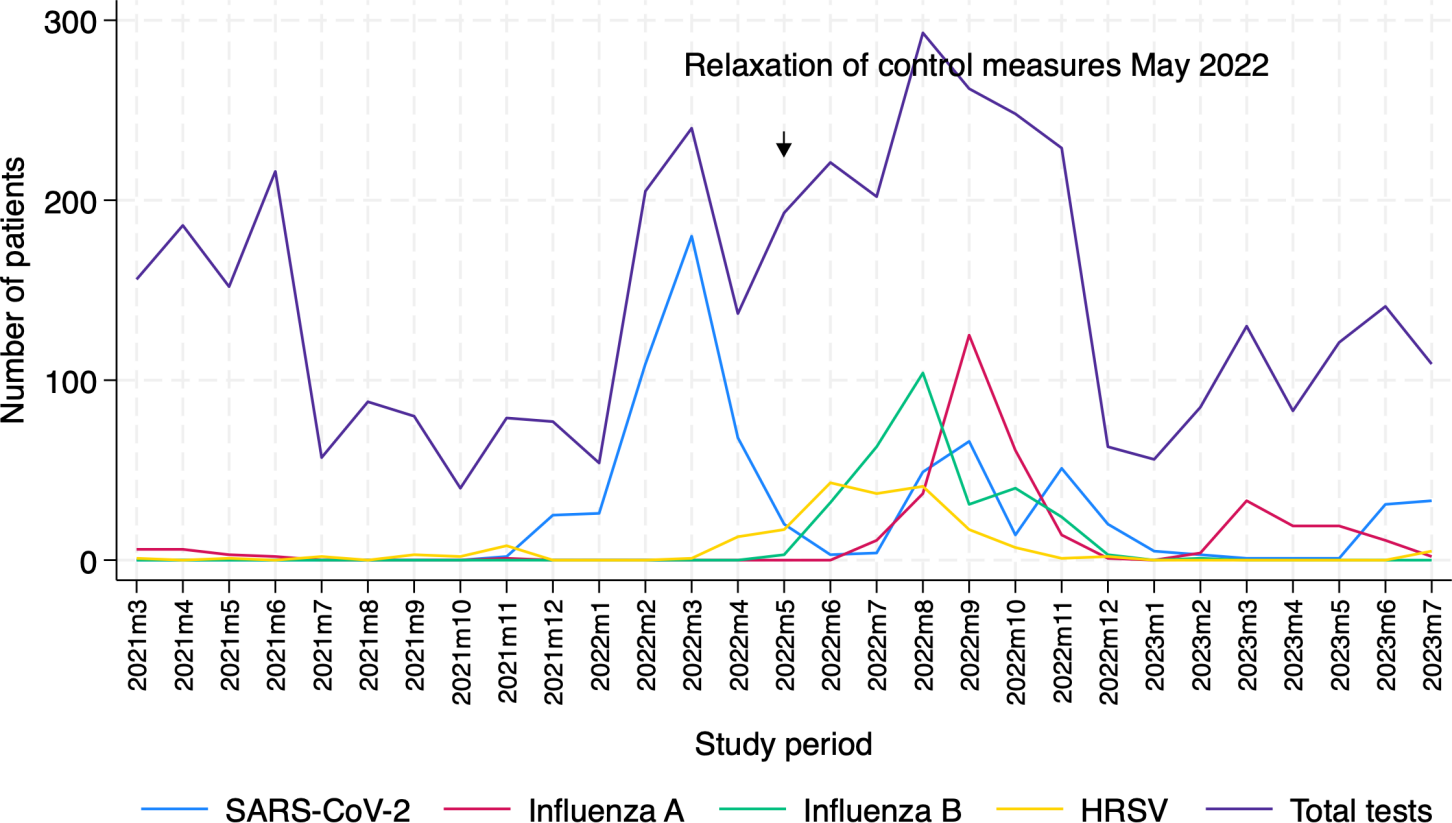
Trends in respiratory virus circulation in the Lao People’s Democratic Republic from March 2021 to July 2023. The downward arrow indicates the timing of the relaxation of control measures in May 2022. hRSV, human respiratory syncytial virus.

1466/4203 (35%) patients tested positive for at least one respiratory virus (table 2). 98 patients had co-detection of two or more respiratory viruses (online supplemental figure 1), of which SARS-CoV-2 and influenza A virus co-detection was the most common. Peaks of hRSV and influenza B virus, followed by influenza A virus, occurred within weeks of easing of lockdown measures, peaking within 3–4 months. Respiratory syncytial virus (RSV) was first detected in May 2022 and peaked in August, while influenza A was first detected again in July 2022 and peaked in September. Influenza B was first detected in August and peaked in October 2022 (figure 2). Most (110/168) cases of hRSV occurred in children under 5 years of age (online supplemental figures 2 and 3).

**Table 2:** Respiratory virus RT-qPCR results on enrolment

Viruses detected, n (%)	Total		LNT		XK		SV		ATP	
	OPD	IPD	OPD	IPD	OPD	IPD	OPD	IPD	OPD	IPD
	n=3281	n=922	n=1276	n=161	n=927	n=597	n=108	n=17	n=669	n=147
SARS-CoV-2	643 (19.6)	69 (7.5)	195 (15.3)	17 (10.6)	256 (20.8)	43 (7.2)	0	0	192 (28.7)	9 (6.1)
Influenza A	293 (8.9)	62 (6.7)	140 (11)	11 (6.8)	70 (5.7)	44 (7.4)	6 (5.6)	0	77 (11.5)	7 (4.8)
Influenza B	281 (8.6)	20 (2.2)	143 (11.2)	1 (0.6)	75 (6.1)	11 (1.8)	0	0	63 (9.4)	8 (5.4)
hRSV	145 (4.4)	56 (6.1)	68 (5.3)	4 (2.5)	33 (2.7)	28 (4.7)	1 (0.9)	1 (5.8)	43 (6.4)	23 (15.6)

At least one virus detected in 1466 (35%) samples; of these, 98 (7%) samples had two or three viruses.

ATP, Attapeu; hRSV, human respiratory syncytial virus; IPD, Inpatient Department; LNT, Luang Namtha; OPD, Outpatient department; RT-qPCR, Real-time quantitative polymerase chain reaction; SV, Salavan; XK, Xiengkhouang.

### Sequencing results

**Figure 3:**
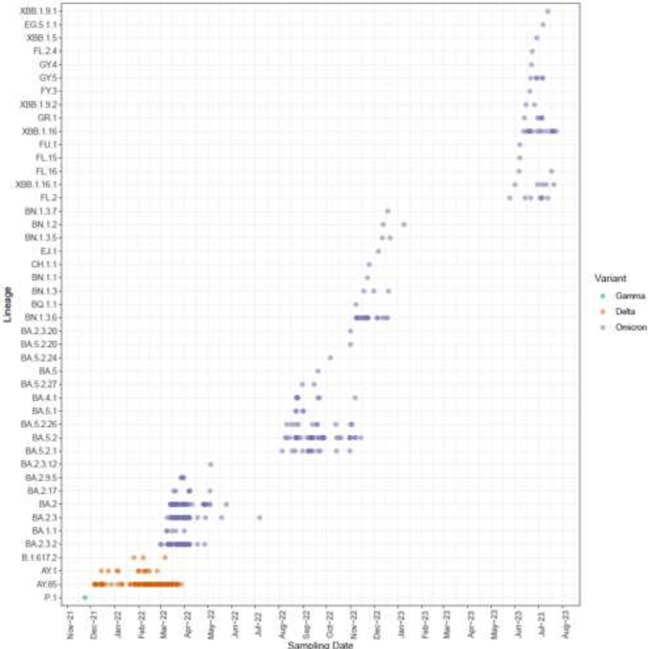
SARS-CoV-2 variants detected during the study. The figure shows the transition from the Gamma to Delta variant in December 2021 and the emergence of the Omicron variant from March 2022.

We submitted 554 and 200 RNA extracts for whole genome sequencing of SARS-CoV-2 and influenza A, respectively; of these, 432 and 114 were sequenced successfully. Results of whole genome sequencing of SARS-CoV-2 and influenza A are shown in figure 3 (online supplemental tables 4 and 5). The Delta variant of SARS-CoV-2 was first detected in December 2021, with Omicron appearing in March 2022, becoming the primary lineage shortly thereafter. Between 23 March and 23 April 2021, we detected influenza A in four patients, among whom three were infected with H3N2 (the virus was not typed for the fourth patient). Influenza A was not detected again until more than 1 year later in July 2022, after which we detected H3N2 every month until November 2022. Influenza A was then not detected again until March 2023, when H1N1 predominated, along with a few cases of H3N2 (online supplemental table 5).

## Discussion

The SARS-CoV-2 infection rate in this study remained low from the start of the outbreak until December 2021, when the number of cases gradually increased and peaked in March 2022, followed by a second peak in September 2022, after the arrival of highly transmissible variants like the Delta variant, closely followed by a succession of the Omicron lineages. The number of cases reported decreased significantly after COVID-19 was no longer considered a Public Health Emergency of International Concern in mid-2022, accompanied by the cessation of large-scale testing, which was no longer mandatory. The lower number of SARS-CoV-2 cases in our study at the beginning of the peak was probably due to people going to designated places for screening and testing rather than hospitals. As observed in many other countries, transmission of common respiratory viruses was interrupted during the SARS-CoV-2 pandemic.^1^ Decreased detection was likely related to the strict enforcement of non-pharmaceutical interventions, including school closures, mask-wearing, physical distancing and travel restrictions. Most patients presenting to hospitals with SARS-CoV-2 in this study had no comorbidities, and their respiratory illness was only mild to moderate. Among the 34 patients in our study who died, 10 had a respiratory virus detected (eight with SARS-CoV-2±other viruses). It was not possible to ascertain if the respiratory viruses were considered the cause of death in individual cases. The increase in COVID-19 cases in Laos occurred only after the national immunisation programme had been implemented, likely obviating the severity of the impact. Laos reported a total of 218 277 cases and 671 deaths from the start of the pandemic until May 2023.^25^

Once lockdown measures relaxed and schools reopened, influenza A, influenza B and hRSV were rapidly detected through our passive surveillance programme after May 2022. Peaks occurred within weeks of initial detection, paralleling regional influenza surveillance data reported by WHO.^11^ Interestingly, once restrictions were eased, hRSV made up the greatest proportion of detected respiratory viral cases in those aged <1 year and 1–4 years, even during a pandemic when SARS-CoV-2 caused greater than 50% of cases among those aged >15 years. This strongly argues for childhood vaccination of hRSV in Laos.

## Conclusions

During lengthy periods of the COVID-19 pandemic, many public health authorities did not perform surveillance for prepandemic respiratory viruses, and many symptomatic infections remained undiagnosed or only syndromically categorised. These data yield valuable insights into true virus prevalence in Laos during and after the COVID-19 pandemic and also elicit interesting questions about the ability of common respiratory viruses to either persist or become extinct during mass and lengthy implementation of public health measures and in competition with emerging pandemic pathogens. Laos successfully avoided widespread community transmission of SARS-CoV-2 until the arrival of new, more transmissible variants, such as Delta and Omicron, by which time there was high uptake of COVID-19 vaccination. Infections with other common respiratory viruses were rare during this period, likely as a consequence of lockdown measures, including school closures, mask-wearing, enhanced hand hygiene and social distancing. However, as soon as lockdown measures eased, influenza A, B and hRSV viruses emerged quickly, peaking within a few weeks.

Our findings show that strengthening routine surveillance systems in low-resource settings could play a crucial role in monitoring the transmission of known and emerging pathogens and in detecting future pandemics.

## Supplementary material

10.1136/bmjopen-2024-098006 online supplemental file 1

## Data Availability

Data are available upon reasonable request.
