# Supplementary material for: Influenza and respiratory syncytial virus dynamics in Lao PDR during the COVID-19 pandemic: a hospital-based surveillance study
Source: BMJ Open. 2025 Sep 4;15(9):e098006. doi: 10.1136/bmjopen-2024-098006 (PMC12414174; doi:10.1136/bmjopen-2024-098006)
Supplement: online supplemental file 1 [file bmjopen-15-9-s001.docx]

**Supplementary material**

# Influenza and respiratory syncytial virus dynamics in Lao P.D.R during the COVID-19 pandemic: a hospital-based surveillance study.

**Supplementary material-1-Laboratory Methods**

*Real-time RT-qPCR assays for virus detection*

Specimens were processed at the central laboratory in Mahosot Hospital, Vientiane. RNA extraction was performed from 200 μL (for 1mL VTM) or 400 μL (for 3 mL VTM) of combined NP and throat swabs in VTM using EZ1 Virus mini kit (Qiagen) eluted in 90 μL. Probe based RT-qPCR were performed as previously described for the detection of SARS-CoV-2 E-gene,^1^ influenza A viruses ^2^ influenza B viruses, ^3^ and human respiratory syncytial virus (hRSV).^4^ Due to a laboratory contamination for the SARS-CoV-2 E-gene RT-qPCR system, the samples collected from November 2021 were tested for SARs-CoV-2 using CDC N1 RT-PCR protocol (US CDC). Each RT-qPCR assay was performed from 5μL RNA using the SuperScript III Platinum One-step RT-PCR system (ThermoFisher) in a final volume and 25μL with 7 μL of primer and probe mix. For each RT-qPCR assays, the corresponding primers and probe had been prepared mixed and freeze dried as previously described^5^ for the primers and probe to be at the adequate concentration in the PCR mix. The thermal profile used was 15 min at 50°C, 2 min at 95°C, 45 cycles of: 15 sec at 95°C and 45 sec at 60°C. A different thermal profile was used for SARS-CoV-2 E-gene RT-qPCR: 10 min at 55°C, 3 min at 94°C, 45 cycles of: 15 sec at 94°C and 30 sec at 58°C. RT-qPCR results with Cq≥37 was confirmed by repeating the corresponding RT-qPCR. RT-qPCR result with Cq<37 was regarded as positive.

Samples found positive for hRSV by RT-qPCR were analyzed to two additional RT-qPCR assays (20), as described above, for the identification of hRSV subtype A and B.

*SARS-CoV-2 Sequencing*

SARS-CoV-2 positive RNA samples with a Ct ≤ 28 were submitted for whole-genome sequencing (WGS) using the Midnight tiling multiplex PCR method.^6,7^ Initially, RNA samples were reverse transcribed by either MultiScribe™ Reverse Transcriptase (Invitrogen™) or SuperScript™ III Reverse Transcriptase (Invitrogen™), or LunaScript® RT SuperMix. Following this, cDNA fragments were amplified to generate 1,200 base pair tiled amplicons across the SARS-CoV-2 genome using either the Phusion High-Fidelity DNA Polymerase (New England Biolabs) with the XGen™ SARS-CoV-2 Midnight Amplicon Panel (IDT), or LunaScript® RT SuperMix, Q5® HS Master Mix with Midnight Primers supplied in the Midnight RT PCR Expansion kit (Oxford Nanopore). Up to 96 samples were barcoded using Oxford Nanopore rapid barcoding kits (SQK-RBK110.96 or SQK-RBK004), pooled together, and then purified by AMPure XP beads (Beckman Coulter). The prepared sequencing libraries were loaded on the MinION Mk1B sequencer (Oxford Nanopore) using a R9.4.1 flow cell. Sequencing was monitored and demultiplexed via MinKNOW software (Oxford Nanopore). The basecalling was done either in real time with the sequencing using MinKNOW software or after sequencing using Guppy software (Oxford Nanopore). The SARS-CoV-2 whole genome sequence was assembled and analysed using the ARTIC workflow as implemented in EPI2ME software (Oxford Nanopore).^8^ The consensus sequences were classified using the Nextclade and Pangolin software packages.

*Influenza A virus amplicon-based sequencing*

The influenza A genomes were sequenced using two methods. The first method was Influenza A amplicon sequencing which was modified from Oxford Nanopore’s Ligation sequencing influenza whole genome (inf_9166_v109_reva_24aug2022) and Zhou (2014) to use the rapid barcoding kit (SQK-RBK110.96) in a similar manner to SARS-CoV-2 sequencing. The second method was OTN hybrid Capture protocol (PraensensBio) whereby influenza A positive RNA samples were converted to double stranded cDNA using NEBNext® Ultra™ II RNA First Strand Synthesis and NEBNext® Ultra™ II Non-Directional RNA Second Strand Synthesis Modules (New England Biolabs).

The tagmented viral cDNA were captured by enrichment probe using Illumina DNA Prep with the Enrichment, (S) Tagmentation kit. The enriched libraries were then amplified, barcoded, and purified using TwistAmp® Basic (TwistDx), Oxford Nanopore’s PCR Barcoding Kit (SQK-PBK004), and KAPA Pure Beads (Roche), respectively. The sequencing libraries prepared from both protocols were then loaded on R9.4.1 flow cells and sequenced on the MinION Mk1B device. The influenza A genome was assembled and typing using either wf-flu from EPI2ME software ^9^ (Oxford Nanopore) or custom pipelines provided by PraesensBio.

**Supplementary material-2-Results**

Supplementary Table 1: Clinical presentation of participants

| Variables | Total | Luang-Namtha | Xiengkhuang | Salavan | Attapeu |
| --- | --- | --- | --- | --- | --- |
|  | N=4,203 | n=1,437 | n=1,825 | n=125 | n=816 |
| Inpatient, n (%) | 922 (21.9) | 161 (11.2) | 597 (32.7) | 17 (13.6) | 147 (18.0) |
| Temp (℃), med (IQR) | 36.7 (36.4-37.2) | 36.5 (36.2-36.8) | 36.7 (36.4-37.3) | 37 (36.7-37.5) | 37 (36.6-37.8) |
| Temp ≥ 37.5 ℃, n (%) | 930 (22.1) | 167 (11.6) | 431 (23.6) | 33 (26.4) | 299 (36.6) |
| HR/min, med. (IQR) | 92 (82-105) | 90 (82-100) | 90 (80-109) | 94 (88-100) | 100 (89-108) |
| RR/min, med (IQR) | 20 (20-26) | 20 (20-24) | 20 (20-25) | 24 (24-28) | 22 (20-30) |
| SBP mmHg, med (IQR) | 117 (107-130) | 118 (108-130) | 120 (110-130) | 111 (100-130) | 111 (100-127) |
| DBP mmHg, med (IQR) | 71 (66-80) | 75 (68-82) | 70 (69-80) | 60 (50-70) | 70 (60-80) |
| SaO2 %, med (IQR) | 98 (96-98) | 98 (97-98) | 97 (56-100) | 96.5 (96-98) | 98 (98-98) |
| Height cm, med (IQR) | 153 (112-160) | 150 (90-160) | 154 (145-161) | 120 (101-150) | 152 (112-160) |
| Weight kg, med (IQR) | 48 (18-58) | 44 (13-56) | 52 (40-60) | 20.5 (16-40) | 45 (17-56) |
| Cough, n (%) |  |  |  |  |  |
| Dry cough | 1,386 (33) | 683 (47.5) | 270 (14.8) | 32 (25.6) | 401 (49.1) |
| Productive cough | 2,595 (61.7) | 703 (48.9) | 1,549 (84.8) | 81 (64.8) | 262 (32.1) |
| haemoptysis | 18 (0.4) | 10 (0.7) | 1 (0.05) | 1 (0.8) | 6 (0.7) |
| Sore throat, n (%) | 2,963 (70.5) | 870 (60.5) | 1,584 (86.7) | 115 (92.0) | 394 (48.3) |
| Runny nose, n (%) | 2,820 (67.1) | 968 (67.4) | 1,235 (67.8) | 98 (78.4) | 519 (63.6) |
| Wheeze, n (%) | 617 (14.8) | 116 (8.1) | 412 (22.5) | 6 (4.8) | 83 (10.2) |
| Dyspnoea, n (%) | 580 (13.8) | 150 (10.4) | 347 (19.0) | 17 (13.6) | 66 (8.1) |
| Chest indrawing, n (%) | 333 (7.9) | 77 (5.4) | 215 (11.9) | 2 (1.6) | 39 (4.8) |
| Chest Pain, n (%) | 875 (20.8) | 72 (5.0) | 697 (38.2) | 6 (4.8) | 100 (12.2) |
| Conjunctivitis, n (%) | 3 (0.07) | 0 | 1 (0.05) | 0 | 2 (0.25) |
| Lymph nodes, n (%) | 75 (1.8) | 5 (0.3) | 67 (3.7) | 1 (0.8) | 2 (0.25) |
| Headache, n (%) | 2,253 (53.6) | 262 (18.2) | 1,464 (80.2) | 105 (84.0) | 422 (51.7) |
| Anosmia, n (%) | 259 (6.2) | 1 (0.07) | 202 (11.1) | 1 (0.8) | 55 (6.7) |
| Ageusia, n (%) | 108 (2.6) | 1 (0.07) | 71 (3.9) | 1 (0.8) | 35 (4.3) |
| Fatigue, n (%) | 1,256 (29.8) | 116 (8.1) | 666 (36.5) | 100 (80.0) | 374 (45.8) |
| Anorexia, n (%) | 331 (7.8) | 8 (0.5) | 2 (0.1) | 22 (17.6) | 299 (36.6) |
| Confusion, n (%) | 9 (0.2) | 3 (0.2) | 4 (0.2) | 1 (0.8) | 1 (0.1) |
| Myalgia, n (%) | 1,255 (29.8) | 147 (10.2) | 708 (38.8) | 47 (37.6) | 353 (43.2) |
| Arthralgia, n (%) | 274 (6.5) | 22 (1.5) | 107 (5.8) | 10 (8.0) | 135 (16.5) |
| Inability to walk, n (%) | 42 (0.9) | 8 (0.5) | 30 (1.6) | 0 | 4 (0.5) |
| Abdominal pain, n (%) | 286 (6.8) | 12 (0.8) | 207 (11.3) | 15 (12.0) | 52 (6.4) |
| Diarrhoea, n (%) | 221 (5.2) | 50 (3.5) | 116 (6.4) | 7 (5.6) | 48 (5.8) |
| Vomiting/nausea, n (%) | 708 (16.8) | 95 (6.6) | 413 (22.6) | 58 (46.4) | 142 (17.4) |
| Skin rash, n (%) | 97 (2.3) | 28 (1.9) | 56 (3.1) | 1 (0.8) | 12 (1.4) |

Supplementary Table 2: Description of qSOFA scores by site

| Variables | Total | Luang-Namtha | Xiengkhuang | Salavan | Attapeu |
| --- | --- | --- | --- | --- | --- |
|  | N=4,203 | n=1,437 | n=1,825 | n=125 | n=816 |
| qSOFA score |  |  |  |  |  |
| qSOFA score=1 | 1,894 (45.1) | 664 (46.2) | 801 (43.8) | 115 (92.0) | 314 (38.5) |
| qSOFA score=2 | 262 (6.2) | 19 (1.3) | 38 (2.1) | 0 | 198 (24.3) |
| qSOFA score=3 | 1 (0.02) | 1 (0.07) | 0 | 0 | 0 |

qSOFA: quick Sequential Organ Failure Assessment. The qSOFA score uses three clinical criteria, each scoring 1 point: altered mental status (Glasgow Coma Scale<15), respiratory rate ≥ 22 breaths per minute, Systolic blood pressure ≤ 100 mmHg.

Supplementary Table 3: Characteristics of patients who died

| No | NP result | Place of death | Gender | Age yr | Vac* | Co-morbidity | BMI | Ward | SaO2 |
| --- | --- | --- | --- | --- | --- | --- | --- | --- | --- |
| 1 | Negative | Died at home | M | 79 | No | CHD, HT | Normal | MED | >90% |
| 2 | Negative | Died at home | F | 60 | No | CHD, HT, CPD | Overweight | IDA | <=90 |
| 3 | Negative | Died at home | M | 35 | No | CPD, Asthma | Underweight | IDA | <=90 |
| 4 | Negative | Died at home | F | 71 | No | CHD, HT, CPD | Normal | IDA | >90% |
| 5 | Negative | Died at home | M | 62 | No | HT, CPD, CKD, Smoker | Normal | IDA | <=90 |
| 6 | Negative | Died at home | F | 66 | No | HT | Obesity I | OPD | >90% |
| 7 | Negative | Died at home | M | 48 | No | No | Normal | IDA | >90% |
| 8 | Negative | Died at home | M | 61 | No | HT, Asthma | Underweight | IDA | <=90 |
| 9 | SARS-CoV-2 | Died at home | M | 78 | No | HT, Asthma | Normal | IDA | <=90 |
| 10 | SARS-CoV-2 | Died at home | F | 58 | No | HT, Asthma | Underweight | IDA | >90% |
| 11 | Negative | Died at home | F | 68 | No | HT | Obesity I | MED | >90% |
| 12 | SARS-CoV-2 & RSV | Died at home | M | 29 | Yes | Smoker | Underweight | IDA | . |
| 13 | Negative | Died at home | M | 27 | No | No | Underweight | IDA | <=90 |
| 14 | Negative | Died at home | M | 52 | Yes | No | Normal | IDA | >90% |
| 15 | Negative | Died at home | M | 68 | No | HT, DM | . | OPD | . |
| 16 | Negative | Died in hospital | F | 62 | No | CHD, CPD | . | IPD | >90% |
| 17 | Negative | Died in hospital | M | 58 | No | CKD | . | IPD | . |
| 18 | Negative | Died at home | F | 50 | No | CHD, CPD, CKD | . | IPD | >90% |
| 19 | SARS-CoV-2 | Died at home | F | 64 | No | No | Normal | OPD | >90% |
| 20 | SARS-CoV-2 | Died in hospital | F | 86 | No | Asthma | Normal | IPD | <=90 |
| 21 | SARS-CoV-2 | Died in hospital | M | 60 | Yes | HIV, CHD | Normal | IPD | . |
| 22 | SARS-CoV-2 & Flu A | Died at home | F | 0.25 | No | No | . | IPD | >90% |
| 23 | SARS-CoV-2 | Died in hospital | F | 48 | No | No | . | IDA | <=90 |
| 24 | Negative | Died at home | M | 86 | No | HT & Hx TB, CPD | . | ICUA | <=90 |
| 25 | Negative | Died in hospital | F | 40 | Yes | CHD, HT | . | ICUA | >90% |
| 26 | Negative | Died at home | F | 75 | Yes | HT & CKD & DM, Smoker | Normal | IPD | >90% |
| 27 | Negative | Died in hospital | M | 57 | No | HT & Smoker | . | IPD | <=90 |
| 28 | Negative | Died in hospital | M | 0.25 | No | No | . | PICU | <=90 |
| 29 | RSV | Died at home | F | 1.6 | No | No | . | PICU | >90% |
| 30 | Flu B | Died at home | M | 52 | Yes | CPD , CKD, DM | Overweight | IPD | >90% |
| 31 | Negative | Died in hospital | M | 13 | No | CHD | Overweight | PICU | >90% |
| 32 | Flu B | Died in hospital | F | 36 | Yes | No | Underweight | ICUA | >90% |
| 33 | Negative | Died in hospital | F | 0.3 | No | Congenital heart Disease | . | PICU | <=90 |
| 34 | Negative | Died in hospital | M | 60 | Yes | No | Normal | ICUA | >90% |

* Received COVID-19 vaccine; Obesity I: BMI=30-34.9

| Variant | Total | Luang-Namtha | Xiengkhuang | Attapeu |
| --- | --- | --- | --- | --- |
|  | n=431 | n=109 | n=173 | n=149 |
| Delta AY.1 | 11 | 0 | 11 | 0 |
| Delta AY.85 | 108 | 17 | 54 | 37 |
| Delta B.1.617.2 | 3 | 0 | 3 | 0 |
| Gamma P.1 | 1 | 0 | 1 | 0 |
| Omicron BA.1.1 | 5 | 0 | 1 | 4 |
| Omicron BA.2 | 53 | 27 | 10 | 16 |
| Omicron BA.2.3 | 41 | 1 | 7 | 33 |
| Omicron BA.2.3.2 | 43 | 0 | 40 | 3 |
| Omicron BA.2.3.12 | 1 | 0 | 0 | 1 |
| Omicron BA.2.3.20 | 1 | 1 | 0 | 0 |
| Omicron BA.2.9.5 | 4 | 0 | 0 | 4 |
| Omicron BA.2.17 | 6 | 0 | 0 | 6 |
| Omicron BA.4.1 | 7 | 4 | 3 | 0 |
| Omicron BA.5 | 1 | 1 | 0 | 0 |
| Omicron BA.5.1 | 4 | 0 | 0 | 4 |
| Omicron BA.5.2 | 37 | 15 | 7 | 15 |
| Omicron BA.5.2.1 | 13 | 2 | 10 | 1 |
| Omicron BA.5.2.20 | 1 | 1 | 0 | 0 |
| Omicron BA.5.2.24 | 1 | 1 | 0 | 0 |
| Omicron BA.5.2.26 | 10 | 4 | 6 | 0 |
| Omicron BA.5.2.27 | 2 | 1 | 1 | 0 |
| Omicron BN.1.1 | 1 | 0 | 0 | 1 |
| Omicron BN.1.2 | 2 | 0 | 2 | 0 |
| Omicron BN.1.3 | 3 | 1 | 0 | 2 |
| Omicron BN.1.3.5 | 2 | 0 | 2 | 0 |
| Omicron BN.1.3.6 | 17 | 9 | 0 | 8 |
| Omicron BN.1.3.7 | 1 | 0 | 1 | 0 |
| Omicron BQ.1.1 | 1 | 0 | 1 | 0 |
| Omicron CH.1.1 | 1 | 0 | 1 | 0 |
| Omicron EG.5.1.1 | 1 | 0 | 1 | 0 |
| Omicron EJ.1 | 1 | 0 | 0 | 1 |
| Omicron FL.2 | 7 | 5 | 0 | 2 |
| Omicron FL.2.4 | 1 | 1 | 0 | 0 |
| Omicron FL.15 | 1 | 1 | 0 | 0 |
| Omicron FL.16 | 2 | 1 | 1 | 0 |
| Omicron FU.1 | 1 | 0 | 0 | 1 |
| Omicron FY.3 | 1 | 1 | 0 | 0 |
| Omicron GR.1 | 5 | 0 | 3 | 2 |
| Omicron GY.4 | 1 | 0 | 1 | 0 |
| Omicron GY.5 | 5 | 0 | 0 | 5 |
| Omicron XBB.1.5 | 1 | 0 | 0 | 1 |
| Omicron XBB.1.9.1 | 1 | 0 | 1 | 0 |
| Omicron XBB.1.9.2 | 2 | 2 | 0 | 0 |
| Omicron XBB.1.16 | 15 | 10 | 3 | 2 |
| Omicron XBB.1.16.1 | 5 | 3 | 2 | 0 |
|  |  |  |  |  |
|  |  |  |  |  |
|  |  |  |  |  |

Supplementary Table 4: Summary of SARS-CoV-2 variants identified during March 2021–July 2023

Supplementary Table 5: Summary of Influenza A typing identified during March 2021–July 2023

| Influenza A typing | Total | Luang-Namtha | Xiengkhuang | Salavan | Attapeu |
| --- | --- | --- | --- | --- | --- |
|  | n=114 | n=48 | n=37 | n=1 | n=28 |
| H1N1 | 17 | 5^*^ | 11 | 0 | 1 |
| H3N2 | 97 | 43 | 26 | 1^*^ | 27 |

^*^ HA type not determined for one sample


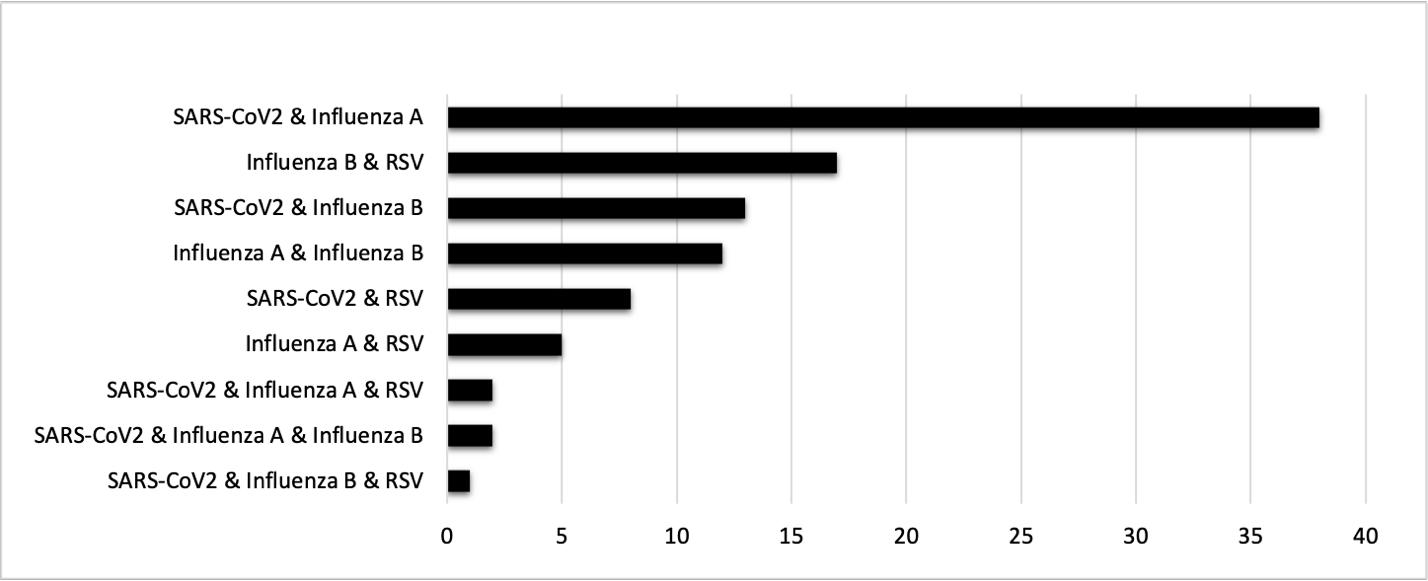


Supplementary Figure 1: Co-detection of multiple pathogens in individual specimens


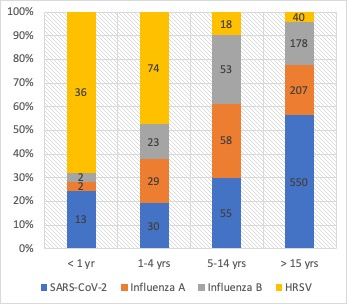


Supplementary Figure 2: Proportion of single infections with SARS-CoV-2, Influenza A, Influenza B, and RSV detected in each age group.


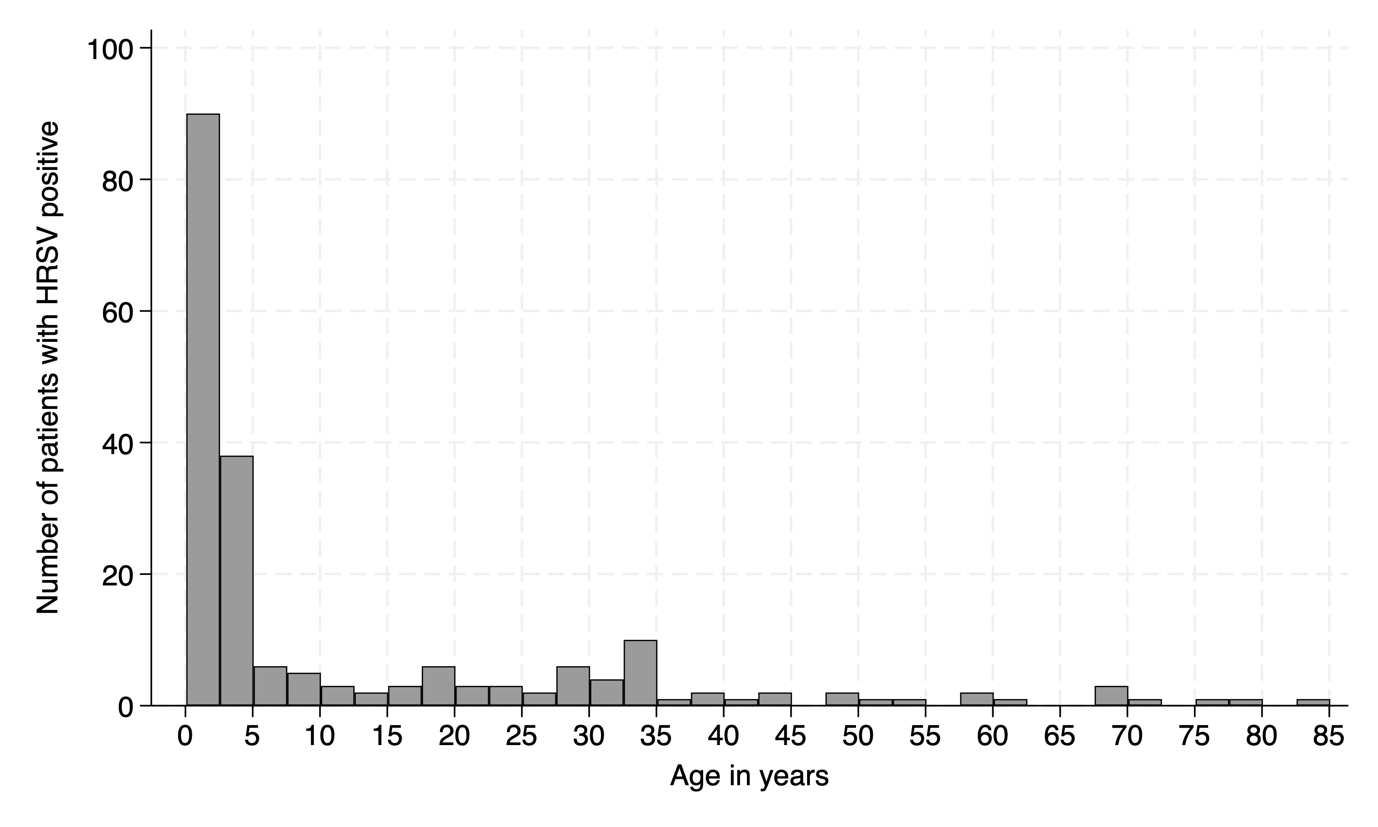


Supplementary Figure 3. Age distribution of patients with RSV

**References**

1. Corman VM, Landt O, Kaiser M, et al. Detection of 2019 novel coronavirus (2019-nCoV) by real-time RT-PCR. *Euro Surveill* 2020; **25**(3).

2. Kim C, Ahmed JA, Eidex RB, et al. Comparison of nasopharyngeal and oropharyngeal swabs for the diagnosis of eight respiratory viruses by real-time reverse transcription-PCR assays. *PLoS One* 2011; **6**(6): e21610.

3. van Elden LJ, Nijhuis M, Schipper P, Schuurman R, van Loon AM. Simultaneous detection of influenza viruses A and B using real-time quantitative PCR. *J Clin Microbiol* 2001; **39**(1): 196-200.

4. Fry AM, Chittaganpitch M, Baggett HC, et al. The burden of hospitalized lower respiratory tract infection due to respiratory syncytial virus in rural Thailand. *PLoS One* 2010; **5**(11): e15098.

5. Thirion L, Dubot-Peres A, Pezzi L, et al. Lyophilized Matrix Containing Ready-to-Use Primers and Probe Solution for Standardization of Real-Time PCR and RT-qPCR Diagnostics in Virology. *Viruses* 2020; **12**(2).

6. Quick J, Grubaugh ND, Pullan ST, et al. Multiplex PCR method for MinION and Illumina sequencing of Zika and other virus genomes directly from clinical samples. *Nat Protoc* 2017; **12**(6): 1261-76.

7. Freed NE, Vlková M, Faisal MB, Silander OK. Rapid and inexpensive whole-genome sequencing of SARS-CoV-2 using 1200 bp tiled amplicons and Oxford Nanopore Rapid Barcoding. *Biol Methods Protoc* 2020; **5**(1): bpaa014.

8. Artic Network SARS-CoV-2 Analysis. 2024.

9. Influenza Typing Workflow. 1.10 ed: EPI2ME Labs; 2024.
