# Supplementary material for: Influenza and respiratory syncytial virus dynamics in Lao PDR during the COVID-19 pandemic: a hospital-based surveillance study
Source: BMJ Open. 2025 Sep 4;15(9):e098006. doi: 10.1136/bmjopen-2024-098006 (PMC12414174; doi:10.1136/bmjopen-2024-098006)
Supplement: Supplementary file 2 [file bmjopen-2024-098006.reviewer_comments.pdf]

PEER REVIEW HISTORY

BMJ Open publishes all reviews undertaken for accepted manuscripts. Reviewers are asked to complete a checklist review form and are provided with free text boxes to elaborate on their assessment. These free text comments are reproduced below.

ARTICLE DETAILS

Title (Provisional)

Influenza and respiratory syncytial virus dynamics in Lao P.D.R during the COVID-19 pandemic: a hospital-based surveillance study.

Authors

Phommasone, Koukeo; Chommanam, Danoy; Christy, Nathaniel C; Yiaye, Touxiong; Phoutthavong, Soulichanya; Keomoukda, Patsalin; Thammavong, Sompong; Bounphiengsy, Thipsavanh; Lathsachack, Thongsavanh; Boutthasavong, Latsaniphone; Vidhamaly, Vayouly; Sibounheuang, Bountoy; Phonemixay, Ooyanong; Panapruksachat, Siribun; Praphasiri, Viladeth; Keomany, Sommay; Chaleunphon, Bounthavy; Douangdala, Phouvieng; Robinson, Matthew T; Batty, Elizabeth M.; Vongsouvath, Manivanh; Wiley, Michael R; Wiley, Jessica D; Chapman, Ryan C; Letizia, Andrew G.; Mayxay, Mayfong; Dubot-Pérès, Audrey; Ashley, Elizabeth A

VERSION 1 - REVIEW

**Reviewer**	**1**
**Name**	**Pierangeli, Alessandra**
**Affiliation**	**University of Rome La Sapienza**
**Date**	**03-Apr-2025**
**COI**	**None**

This prospective, case-based surveillance for respiratory virus infections was conducted in four provincial hospitals in Lao P.D.R. between March 2021 and July 2023. Respiratory infection testing for SARS-CoV-2, influenza A and B, and respiratory syncytial virus was performed in around 4000 patients of all ages by real-time RT-qPCR at the central laboratory in Mahosot Hospital, Vientiane. Whole genome sequencing was also performed for SARS-CoV-2 and influenza A virus.

Overall, the study's description of the epidemiology of SARS-CoV-2 is an interesting contribution, as few data exist for this region. Other virological findings are also interesting for the post-pandemic global epidemiology of influenza and RSV, but should be discussed in

the context of the global post-pandemic scenario. The clinical description of the cases is detailed.

Several points should be modified.

The title "Respiratory virus dynamics in Lao P.D.R. during the COVID 19 pandemic..." should be changed to "Influenza and respiratory syncytial virus dynamics in ....".

Introduction

Lines 100-102: “The first cases of laboratory confirmed COVID-19 in Laos were reported on 23 March 2020. Following a nationwide lockdown from early April 2020, a national seroprevalence study conducted in August-September 2020 confirmed negligible SARS-CoV-2 circulation in the community at that time.” It should be reported whether molecular tests for SARS-CoV-2 were available in the first year of Covid-19 pandemic period, i.e. before the start of this study.

Methods

page 8, lines 146-147: "Details of the real-time RT-qPCR and whole genome sequencing methods for SARS-CoV-2 and influenza A virus can be found in the supplementary material". In the main text of the Methods, it should be stated that the real-time RT-qPCR reactions were developed in-house according to published protocols. In addition, it should be indicated whether the whole genome sequencing was carried out on the diagnostic sample and in which laboratory it was performed.

Results

At the beginning of the "Virus Detection" section, I would suggest that the exact number of patients tested be reported as follows: "A total of 1,466/xxxx (35%) patients tested positive...". In this regard, in the abstract, it is reported “4,203 patients recruited”

It should be specified whether the patients were tested only once or whether more than one sample per patient was included in the study group.

Lines 195-196: Results of Whole Genome Sequencing of SARS-CoV-2 and Influenza A are shown in Figure 3, Table 3 and Supplementary Table 4.” There is no Table 3 in the text. It should be mentioned how many samples were successfully sequenced.

Discussion

At the beginning of the Discussion section (line 214), the sentence: "The COVID-19 infection rate" should read: "In this study, the SARS-CoV-2 infection rate".

Line 220: "The lower number of SARS-CoV-2 cases in our study at the beginning of the peak was probably due to ..." It is not clear whether the authors compared the cases in this study with other national data or with the worldwide high incidence of SARS-CoV-2 infection. It should be specified whether quantitative data from Laos (ref 21) can be compared with those presented here; otherwise, it would be better to change the sentence in "The

relatively low number of SARS-CoV-2 cases in our study..." lines 215-216: "...July 2021, when the number of cases gradually increased and peaked in December 2021, followed by a second peak in April 2022, after the emergence of highly transmissible variants such as the Delta variant, closely followed by a succession of Omicron lineages." This sentence is not clear, and only a peak in March 2022 is visible in Figure 3.

Figure legends are provided for Figures 1 and 2, but they should be more explanatory. Figure 3 does not have a legend.

**Reviewer**	**2**
**Name**	**Nazneen, Arifa**
**Affiliation**	**International Centre for Diarrhoeal Disease Research Bangladesh, Infectious Diseases Division**
**Date**	**07-May-2025**
**COI**	**None**

Review comments on the manuscript titled: Respiratory virus dynamics in Lao P.D.R during the COVID-19 pandemic: a hospital-based surveillance study.

Abstract

The format of the structured abstract, as stated in the author guidelines of BMJ Open, was not followed.

Line 48: “At least two viruses were detected in 98 patients.” The sentence is not complete. The percentage also needs to be mentioned here.

Line 49: “hRSV were detected in very low numbers of patients”-here you need to mention the statistics.

Research in context

Line 55 and 64: “non-pharmaceutical intervention” is mentioned, but not stated for what? It was stated below (the measures), but here, good to say: non-pharmaceutical intervention for infection control.

Line 71: “Our study shows that strengthening routine surveillance systems in low resource settings,” But in the manuscript, it was not mentioned that the study sites were in low resource settings.

Background

Overall informative, however, it would be good to have data on pre-COVID viral dynamics with proper citations in the background.

Line 84, 96, 109, and 110: Citation missed.

Sentences were sometimes too long and complex.

Methods

The method needs revision. Study design not mentioned. How were the study sites selected? Why?

Why were the tertiary care hospitals not selected? As mentioned the bed numbers, the hospitals seem to be the primary health care facilities.

The sampling technique was a convenience sampling approach (line 239). Why?

All enrolled patients were visited at the outpatient clinic? Or both out and inpatient? What was the enrollment strategy?

Have all patients been approached? How many refusals? What was the main reason for refusal? Have we missed any specific group?

Who did the interviews, and who collected samples/specimens? Provided standard training or not? Any SOP?

Line123: Figure 1 (?Map), legend missing

The sample size is not mentioned anywhere. How are sample sizes calculated?

Line 127: Mentioned written consent; nonetheless, more information on consent, including “Assent”, should be mentioned.

Data analysis: More in-depth analysis can be added, including comparing the sites. Sociodemographic relation. Symptom distributions, etc.

Ethics: Need to mention the process, types, and contents of the consents. How was data safety, confidentiality, and anonymity maintained?

Results

Participants' distributions per site were of different values

Line 164: “patients had mild or moderate disease (qSOFA < 2).” You mentioned qSOFA, but it would be good to have more information on qSOFA. What is it? Why is it used here? What are the scores? The scoring system?

Line 167: What does it mean? “(data not shown)”

Line 175: “Using qSOFA criteria, 263 (6%) participants met the criteria for suspected sepsis”… what were the criteria?

Line: 176, 177, 185, and 191- mention the percentages

Patient

Tables and Figures:

Table legends should be added, and they should explain all abbreviations used, including raw or column percentages. Captions of figures 1 and 2 are missing.

Line 204: “N=4,203” total of 4 sites, not the total population, so “N” would be “n”

Discussion

The discussion part should be more robust. Discussion points need to be supported by citations.

Data collection and testing-related limitations can be added, if any.

Conclusion

Line 247: “…prior to …” does prior to the COVID pandemic mean? Is it true?

Line 248: What do you mean by true virus?

Lines 254 and 255: “likely as a consequence of lockdown measures, including school closures, mask wearing, enhanced hand hygiene, and social distancing”. can be replaced by “Control measures”

The conclusion also needs revision: Include the possible study impact, way forward, or recommendations.

References

Please recheck the references and follow the BMJ recommended reference style.

Supplementary materials

STROBE checklist not included. The study protocol can be shared. References should be well formatted.

Overall

Please check the English language, use of punctuation

VERSION 1 - AUTHOR RESPONSE

Reviewer: 1

Dr. Alessandra Pierangeli, University of Rome La Sapienza

Comments to the Author:

This prospective, case-based surveillance for respiratory virus infections was conducted in four provincial hospitals in Lao P.D.R. between March 2021 and July 2023. Respiratory infection testing for SARS-CoV-2, influenza A and B, and respiratory syncytial virus was

performed in around 4000 patients of all ages by real-time RT-qPCR at the central laboratory in Mahosot Hospital, Vientiane. Whole genome sequencing was also performed for SARS-CoV-2 and influenza A virus.

Overall, the study's description of the epidemiology of SARS-CoV-2 is an interesting contribution, as few data exist for this region. Other virological findings are also interesting for the post-pandemic global epidemiology of influenza and RSV, but should be discussed in the context of the global post-pandemic scenario. The clinical description of the cases is detailed.

Several points should be modified.

The title "Respiratory virus dynamics in Lao P.D.R. during the COVID 19 pandemic..." should be changed to "Influenza and respiratory syncytial virus dynamics in ....".

Introduction

Response

Thank you for this comment. The title has been changed as suggested

Lines 100-102: "The first cases of laboratory confirmed COVID-19 in Laos were reported on 23 March 2020. Following a nationwide lockdown from early April 2020, a national seroprevalence study conducted in August-September 2020 confirmed negligible SARS-CoV-2 circulation in the community at that time." It should be reported whether molecular tests for SARS-CoV-2 were available in the first year of Covid-19 pandemic period, i.e. before the start of this study.

Response: we added: PCR testing was established in Laos in February 2020.

Methods

page 8, lines 146-147: "Details of the real-time RT-qPCR and whole genome sequencing methods for SARS-CoV-2 and influenza A virus can be found in the supplementary material". In the main text of the Methods, it should be stated that the real-time RT-qPCR reactions were developed in-house according to published protocols. In addition, it should be indicated whether the whole genome sequencing was carried out on the diagnostic sample and in which laboratory it was performed.

Response:

In-house PCR protocol generally refers to PCR system entirely designed from scratch (including primers and probes sequences) by the laboratory. However, in our case we used published PCR system, we just adapted slightly the PCR conditions. Therefore, to avoid the confusion we didn't use the term "in-house" but modified the sentence as below to take into account the reviewer comment:

"RNA extraction was performed using the EZ1 Virus mini kit (Qiagen) then submitted to probe-based real-time RT-PCR (RT-qPCR) for the detection of SARS-CoV-2[21] influenza A virus[22], influenza B virus[23] and hRSV[24] using conditions adapted from the corresponding publications. SARS-CoV-2 and Influenza A virus whole genome sequencing were also performed at the same laboratory on the samples found positive by the corresponding RT-qPCR with a Ct$\leq$ 28. Details of the RT-qPCR and whole genome sequencing methods for SARS-CoV-2 and influenza A virus can be found in the supplementary material."

Results

At the beginning of the "Virus Detection" section, I would suggest that the exact number of patients tested be reported as follows: "A total of 1,466/xxxx (35%) patients tested positive...". In this regard, in the abstract, it is reported "4,203 patients recruited"

It should be specified whether the patients were tested only once or whether more than one sample per patient was included in the study group.

Response: Thank you and we have added 1,466/4,203. Patients were only tested once which we think most readers will assume.

Lines 195-196: Results of Whole Genome Sequencing of SARS-CoV-2 and Influenza A are shown in Figure 3, Table 3 and Supplementary Table 4." There is no Table 3 in the text. It should be mentioned how many samples were successfully sequenced.

Response: Thank you for pointing this out. The sentence now refers to Supplementary Table 5

We also added: "we submitted 554 and 200 RNA extracts for whole genome sequencing of SARS-CoV-2 and Influenza A, respectively. Of those, 431and 114 were sequenced successfully."

Discussion

At the beginning of the Discussion section (line 214), the sentence: "The COVID-19 infection rate" should read: "In this study, the SARS-CoV-2 infection rate".

Response: This sentence has been edited to read: “The SARS-CoV-2 infection rate in this study remained low from the start of the outbreak……”

Line 220: "The lower number of SARS-CoV-2 cases in our study at the beginning of the peak was probably due to ..." It is not clear whether the authors compared the cases in this study with other national data or with the worldwide high incidence of SARS-CoV-2 infection. It should be specified whether quantitative data from Laos (ref 21) can be compared with those presented here; otherwise, it would be better to change the sentence in "The relatively low number of SARS-CoV-2 cases in our study..." lines 215-216: "...July 2021, when the number of cases gradually increased and peaked in December 2021, followed by a second peak in April 2022, after the emergence of highly transmissible variants such as the Delta variant, closely followed by a succession of Omicron lineages." This sentence is not clear, and only a peak in March 2022 is visible in Figure 3.

Figure legends are provided for Figures 1 and 2, but they should be more explanatory. Figure 3 does not have a legend.

Response:

Thank you very much. We changed accordingly: “The SARS-CoV-2 infection rate in this study remained low from the start of the outbreak until December 2021, when the number of cases gradually increased and peaked in March 2022, followed by a second peak in September 2022, after the arrival of highly transmissible variants like the Delta variant, closely followed by a succession of Omicron lineages.”

We have revised the legends for the figures as follows

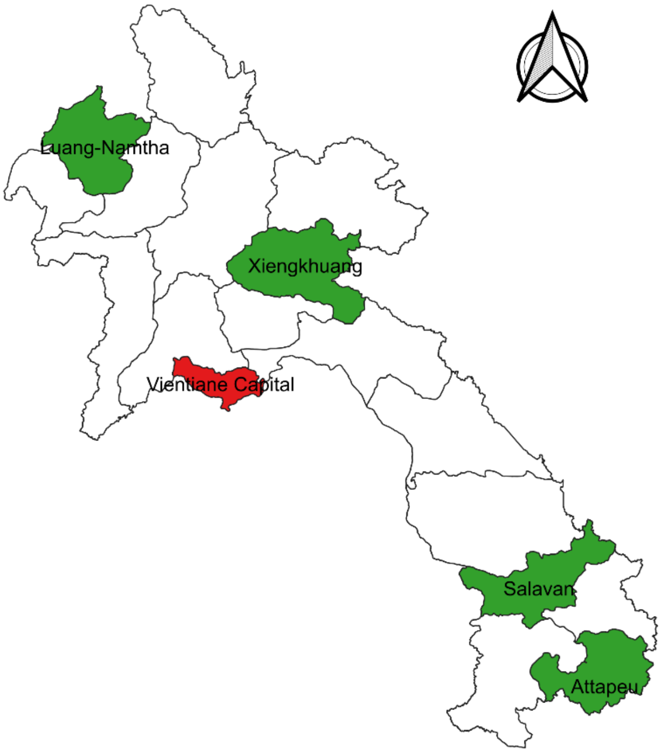

Figure 1: Map of Lao PDR indicating study sites in green and Vientiane Capital, where the central laboratory is located, in red

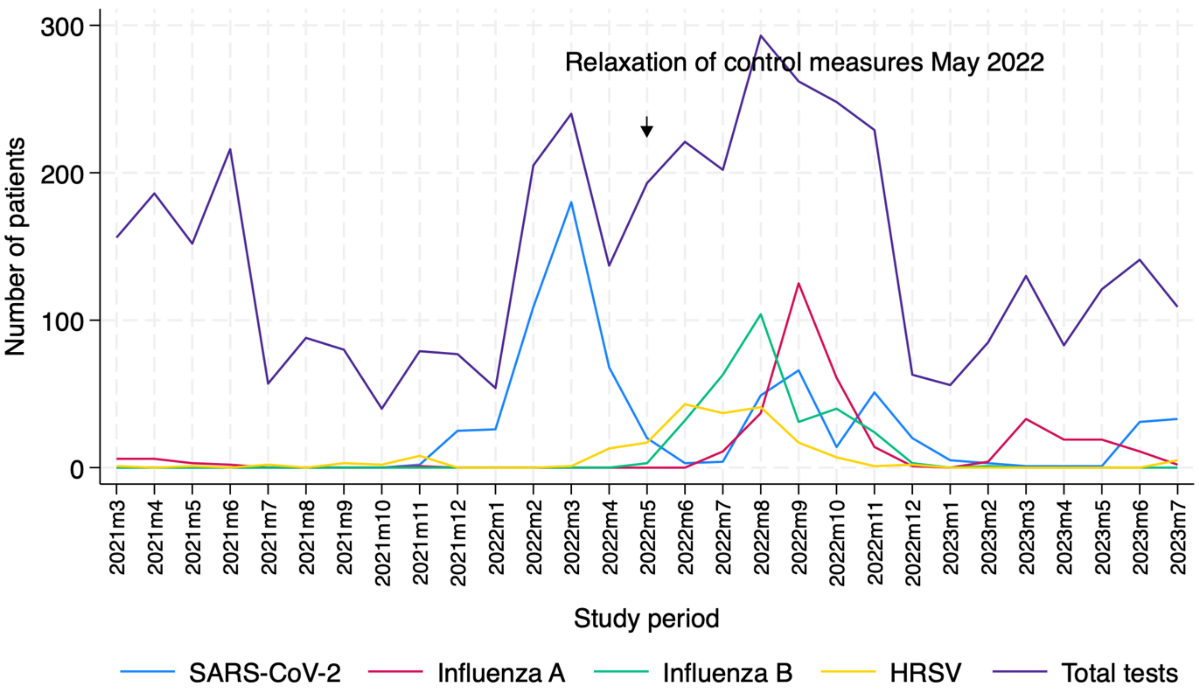

Footnote: Downward arrow indicates the timing of relaxation of control measures (May 2022)

Figure 2: Trends in respiratory virus circulation in Lao PDR from March 2021 to July 2023

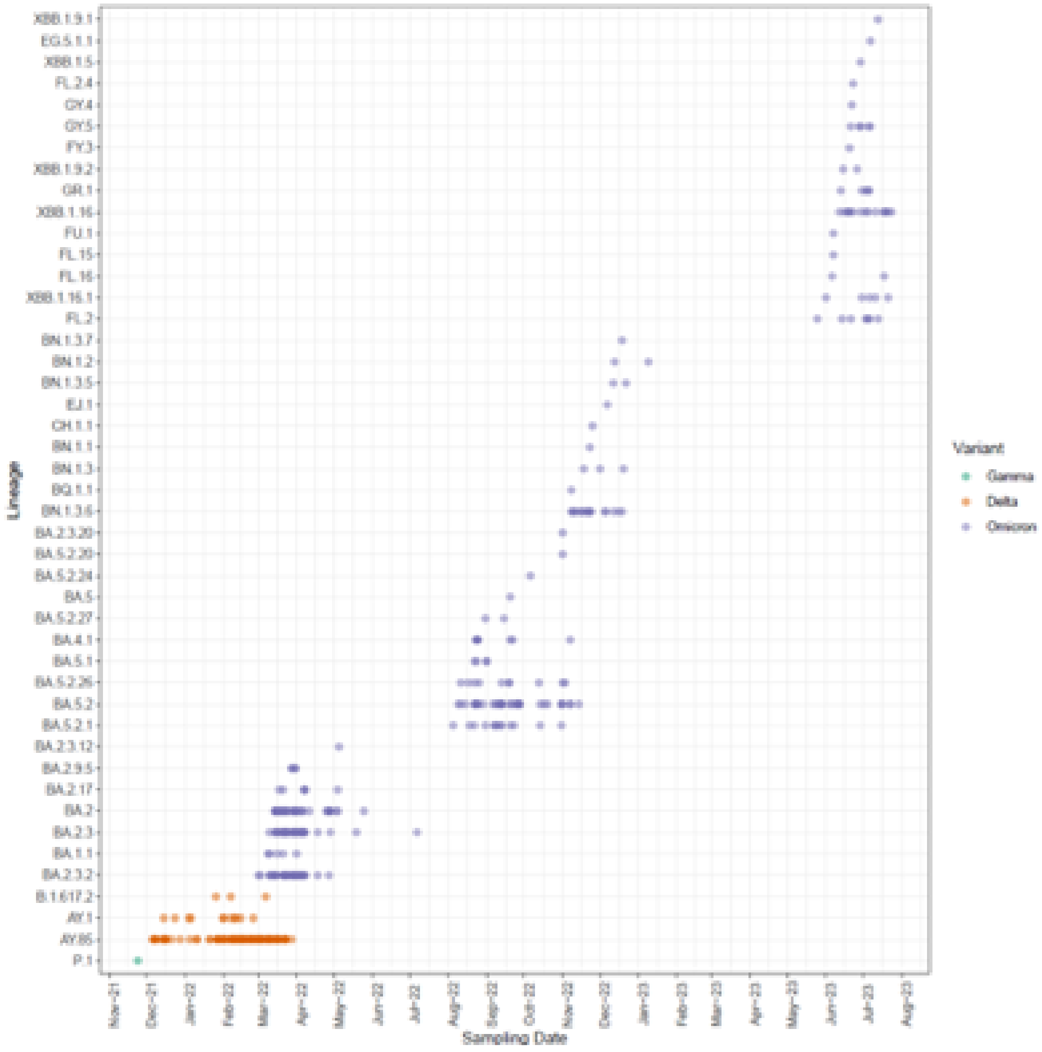

Figure 3: SARS-CoV-2 variants detected during the study, showing the transition from Gamma to Delta variant in December 2021 and the emergence of Omicron variants from March 2022

Reviewer: 2

Dr. Arifa Nazneen, International Centre for Diarrhoeal Disease Research Bangladesh

Comments to the Author:

Review comments on the manuscript titled: Respiratory virus dynamics in Lao P.D.R during the COVID-19 pandemic: a hospital-based surveillance study.

Abstract

The format of the structured abstract, as stated in the author guidelines of BMJ Open, was not followed.

Response: our paper was transferred from BMJ Global Health which has a different format. We reformatted the abstract according.

Line 48: “At least two viruses were detected in 98 patients.” The sentence is not complete. The percentage also needs to be mentioned here.

Response: This has been edited as follows:

“Ninety-eight (2.3%) patients were diagnosed with probable coinfection, with at least two viruses detected.

Line 49: “hRSV were detected in very low numbers of patients”-here you need to mention the statistics.

Response: thank you very much. We changed as follows: Influenza A, Influenza B, and hRSV were detected in 12, 6, and 23 patients, respectively, out of 1,767 recruited patients from the start of the study until the relaxation of control measures in May 2022.

Research in context

Line 55 and 64: “non-pharmaceutical intervention” is mentioned, but not stated for what? It was stated below (the measures), but here, good to say: non-pharmaceutical intervention for infection control.

Response: Thank you very much for the comment. We added as suggested: During the COVID-19 pandemic in Lao PDR, few respiratory viruses were detected by passive surveillance until the relaxation of non-pharmaceutical interventions implemented for infection control.

Line 71: "Our study shows that strengthening routine surveillance systems in low resource settings," But in the manuscript, it was not mentioned that the study sites were in low resource settings.

Response: Laos is a lower middle-income country as defined by the World Bank. We intended to indicate the country more generally.

Background

Overall informative, however, it would be good to have data on pre-COVID viral dynamics with proper citations in the background.

Line 84, 96, 109, and 110: Citation missed.

Sentences were sometimes too long and complex.

Response: We have some pre-pandemic data from a case-control study of acute respiratory infection before the outbreak of COVID-19. This is referenced on line 86-90"Although respiratory infection data are limited in Lao PDR, a previous study of patients with symptoms of acute respiratory infection and age- and sex-matched community controls between June 2019 and June 2020 at one site in northeastern Laos attributed the etiology of symptoms to influenza B virus, influenza A virus, human metapneumovirus (hMPV), and hRSV in 17.8%, 17.2%, 7.5%, and 6.5% of participants, respectively.[15]

Methods

The method needs revision. Study design not mentioned. How were the study sites selected? Why?

Why were the tertiary care hospitals not selected? As mentioned the bed numbers, the hospitals seem to be the primary health care facilities.

The sampling technique was a convenience sampling approach (line 239). Why?

All enrolled patients were visited at the outpatient clinic? Or both out and inpatient? What was the enrollment strategy?

Have all patients been approached? How many refusals? What was the main reason for refusal? Have we missed any specific group?

Who did the interviews, and who collected samples/specimens? Provided standard training or not? Any SOP?

Response:

We have clarified the approach to enrollment as follows:

This surveillance study was conducted at four provincial hospitals, which serve as secondary care facilities: Luang Namtha (LNT) Provincial Hospital, a 60-bedded hospital close to the Laos-China border; Xiengkhuang (XK) Provincial Hospital (100 beds) in northeastern Laos; and Salavan (SV) Provincial Hospital (70 beds) and Attapeu (ATP) Provincial Hospital (100 beds), both located in southern provinces, close to Laos-Cambodia and Laos-Vietnam borders (Figure 1). This study was designed as prospective case-based surveillance of outpatients and inpatients and formal sample size calculation was not performed.

We have clarified the recruitment times and that we employed research teams (2 staff at each site)

Line 128-129 “Symptoms, medical history, vital signs and physical examination findings were documented by trained research staff”

Line123: Figure 1 (?Map), legend missing

Response

Please see above in response to editorial comments

The sample size is not mentioned anywhere. How are sample sizes calculated?

Response

This has been incorporated into our updated paragraph explaining the approach to recruitment.

Our study was designed as a prospective surveillance and our teams recruited patients during daytime working hours on weekdays. However they could still include patients meeting our case definition who had been hospitalised overnight.

Line 127: Mentioned written consent; nonetheless, more information on consent, including “Assent”, should be mentioned.

Response

Thank you for pointing out this omission. We have added the following sentence:

Written informed consent was obtained for patients aged 15 years and above, with assent obtained from children aged between 12 and 15 years.

Data analysis: More in-depth analysis can be added, including comparing the sites. Sociodemographic relation. Symptom distributions, etc.

Response

Thank you for this comment. We did not design the study to compare participant characteristics across the sites and we respectfully prefer not to add this. There may be biases which would make any observation difficult to interpret such as our convenience sampling approach across the different hospital departments, different approaches to screening and triaging patients suspected of having COVID by the different provincial health authorities, staggered immunization deployment etc. for which we have incomplete information.

Ethics: Need to mention the process, types, and contents of the consents. How was data safety, confidentiality, and anonymity maintained?

response:

We added more on the consent above as well as another sentence in the methods on confidentiality

"Patient data were pseudonymized with each patient given a study ID which was entered on the case report form. All paper records were kept securely in a locked filing cabinet when not in use. No participant identifiable information was entered into the study database"

Participants' distributions per site were of different values

Response: as this is a surveillance study, the number of patients recruited varied across sites. This variation was due to differences in the number of patients presenting with acute respiratory symptoms, which in turn depends on the population size served by each hospital.

Line 164: "patients had mild or moderate disease (qSOFA < 2)." You mentioned qSOFA, but it would be good to have more information on qSOFA. What is it? Why is it used here? What are the scores? The scoring system?

Response:

The quick Sequential Organ Failure Assessment (qSOFA) was also used to identify patients with suspected infection who may be at risk of clinical deterioration. This universal screening tool is composed of three criteria: altered mental status, respiratory rate ≥ 22 breaths per minute, and systolic blood pressure ≤ 100 mmHg. A score of two or more suggests a higher risk of mortality.

. We have added a reference to the score definition.

Line 167: What does it mean? "(data not shown)"

Response: This means the data are not published

Line 175: "Using qSOFA criteria, 263 (6%) participants met the criteria for suspected sepsis"… what were the criteria?

Response: This means qSOFA criteria. We have substituted "score" for the first appearance of the word "criteria" as follows

Using the qSOFA score, 263 (6%) participants met the criteria for suspected sepsis"

Line: 176, 177, 185, and 191- mention the percentages

Response

These have been added as follows: Among 3939 patients, 108 (2.7%) presented with oxygen saturations of 90% or lower on room air. Using qSOFA score, 263 (6%) participants met the criteria for suspected sepsis and 45 (1%) patients (including 31 children) were admitted to the intensive care unit. Thirty-four patients (0.8%), including18 males, died with a median age of 58 years ranging from 3 months to 86 years. Of those, 12 (0.3%) patients died in hospital and 22 (0.5%) patients died at home within 28 days of hospital discharge.

Tables and Figures:

Table legends should be added, and they should explain all abbreviations used, including raw

or column percentages. Captions of figures 1 and 2 are missing.

Response

Table and figure legends have all been added and abbreviations defined.

Line 204: “N=4,203” total of 4 sites, not the total population, so “N” would be “n”

Response: thank you very much. We corrected and added as suggested.

Discussion

The discussion part should be more robust. Discussion points need to be supported by citations.

Data collection and testing-related limitations can be added, if any.

Response

Thank you for the comments. As this was prospective surveillance conducted during the pandemic rather than having an experimental design we can only speculate on the association between our findings and the implementation of NPI, hence we have to be cautious about inferring causation. We included a paragraph on the main limitations of our study including the failure to test for bacterial causes of pneumonia.

Line 222 we have added more citations from studies in other countries which made similar observations

Conclusion

Line 247: “…prior to …” does prior to the COVID pandemic mean? Is it true?

Line 248: What do you mean by true virus?

Response

This refers to the data we have pre-pandemic. This sentence has been revised for clarity

“These data yield valuable insights into true virus prevalence in Laos during and after the COVID-19 pandemic, but also elicit interesting questions into the ability of common respiratory

viruses to either persist or become extinct during mass and lengthy implementation of public health measures and competition with emerging pandemic pathogens”

Lines 254 and 255: “likely as a consequence of lockdown measures, including school closures, mask wearing, enhanced hand hygiene, and social distancing”. can be replaced by “Control measures”

Response

We would like to keep the various measures listed separately if possible.

The conclusion also needs revision: Include the possible study impact, way forward, or recommendations.

Response

Thank you for these comments. We have added a concluding sentence as follows

“Our findings show that strengthening routine surveillance systems in low resource settings could play a crucial role in monitoring the transmission of known and emerging pathogens and in detecting future pandemics.”

References

Please recheck the references and follow the BMJ recommended reference style.

Response

References have been checked

Supplementary materials

STROBE checklist not included. The study protocol can be shared. References should be well formatted.

Response

The STROBE checklist has been added. We have not included the protocol, although this can be done if this is an editorial requirement.

Overall

Please check the English language, use of punctuation

Response

We have proofread the manuscript, made edits, and submitted it with highlighted changes

VERSION 2 - REVIEW

**Reviewer**	**1**
**Name**	**Pierangeli, Alessandra**
**Affiliation**	**University of Rome La Sapienza**
**Date**	**11-Aug-2025**
**COI**	

The authors addressed my comments and improved the paper.
